# Reduction of needlestick injuries in healthcare personnel at a university hospital using safety devices

**DOI:** 10.1186/1745-6673-8-20

**Published:** 2013-07-29

**Authors:** Cornelia Hoffmann, Lutz Buchholz, Paul Schnitzler

**Affiliations:** 1 Occupational Health Service, University Hospital Heidelberg, Heidelberg, Germany; 2 Am Büchsenackerhang 65/1, Heidelberg, Germany; 3 Department of Infectious Diseases, Virology, University of Heidelberg, Heidelberg, 69120, Germany

**Keywords:** Bloodborne infection, Needlestick injury, Safety device, Healthcare personnel, Occupational exposure

## Abstract

**Background:**

Healthcare personnel (HCP) is exposed to bloodborne pathogens through occupational risk factors. The objective of this study was to compare the incidence of needlestick injuries (NSIs) before and after the introduction of safety devices in all departments of our hospital.

**Methods:**

Data was extracted from mandatory needlestick report forms of the hospital’s Occupational Health Service. Serological results of patients and healthcare personnel (HCP) were reviewed in the laboratory information system.

**Results:**

In 2007, the year before the introduction of safety devices, 448 needlestick injuries were self-reported, corresponding to an annual rate of 69.0 NSIs per 1 000 full-time HCP. The highest incidence was observed among medical staff in the surgery department and internal medicine with 152 (33.9%) and 79 (17.6%) NSIs, respectively. Of all occupational groups, nurses (36.2%) had the highest risk to sustain NSIs. In 2008 safety devices were introduced across the hospital, e.g. peripheral venous catheter, hypodermic needle and stapling system for wound sealing providing active or passive protection. In 2009, the year after introduction of safety devices, only 350 NSIs were reported, the annual rate of NSIs decreased to 52.4 per 1 000 full-time HCP. Thus an overall reduction of 21.9% for NSIs was achieved when safer devices were applied. The number of NSIs was reduced by even 50% for blood withdrawal, for use of peripheral venous catheters and application of hypodermic needles.

**Conclusion:**

The application of safety devices led to a reduction of NSIs and significantly reduces the risk of bloodborne infections.

## Introduction

In Germany, about 500 000 needlestick injuries (NSIs) occur annually among healthcare personnel (HCP), including injuries from syringes, sewing needles, and other sharp devices [1,2]. Globally, more than 35 million HCP face the risk of sustaining a percutaneous injury with a contaminated sharp instrument every year [3]. The annual number of injuries per healthcare personnel varies from 0.2 – 4.7/year [4]. Worldwide, the number of HCP annually exposed to sharps injuries contaminated with hepatitis B virus (HBV), hepatitis C virus (HCV) or human immunodeficiency virus (HIV), is estimated at 2.1 million, 926 000, and 327 000, respectively. Each year, 66 000 HBV, 16 000 HCV and 1 000 HIV infections were estimated to occur among HCP worldwide due to their occupational exposure to percutaneous injuries [4].

The risk of transmission of infection via NSI is reported to be 6 – 30% for hepatitis B (without vaccination), 2 – 3% for hepatitis C and 0.3% for HIV [1,5]. Vaccination is one of the best ways to protect HCP from infections, but vaccination is only available against hepatitis B. In order to decrease the risk of preventable infections, complete coverage of vaccination against hepatitis B should be achieved. As there is still no vaccine available against hepatitis C and HIV, preventive measures against NSIs is of great importance. Although no available prophylaxis exists for hepatitis C, it is crucial to identify HCV exposure and infection in healthcare settings and to consequently propose early treatment when transmission occurs. In contrast to HCV, HIV highlights a bloodborne disease that can be reduced significantly through timely administration of post exposure prophylaxis (PEP). Reporting occupational NSIs directly to the occupational health service is of major importance preventing transmission of bloodborne diseases. Furthermore, reporting facilitates appropriate counselling and timely post exposure interventions.

In Germany, the “Technical Rule 250 – Biological Agents in Healthcare and Welfare Facilities” requires that spike, sharp, or breakable devices should be replaced by suitable devices or methods, which have no or low risk for NSIs [6]. This is mandatory for the treatment of patients infected with biosafety level 3 organisms (e.g. HBV, HCV, HIV). Safety devices should be used for all procedures if infection relevant body fluids could be transmitted. Strategies are available to prevent infections due to NSI, including education of HCP and reduction of invasive procedures. The use of safety devices can prevent injuries from sharp objects and reduce patients’ risk of exposure to the blood of injured personnel [7]. Nevertheless, the implementation of safety devices in Germany has partly failed due to higher costs.

The aim of this study was to evaluate the prevalence of NSIs among HCP in a university hospital in Southwestern Germany. Furthermore, the study aimed to analyse the reduction of NSIs after implementation of safety devices.

## Methods

Needlestick injuries (NSIs) are defined as percutaneous injuries with sharp objects contaminated with blood or other body fluids. The University Hospital Heidelberg is a 2 000 beds tertiary care hospital and treated 199 726 patients in the year 2007, 213 847 patients in 2009, which corresponds to an increase of 6.6% in the number of patients. In 2007, 6493 full-time healthcare personnel (HCP) were employed, 6683 full-time medical staff was employed in 2009, corresponding to an increase of 2.8% HCP at the University Hospital Heidelberg.

Serological data was extracted for from the laboratory information system and data on NSIs from the mandatory NSI report forms. These forms are available at our university hospital since 1997 and are collected at the Occupational Health Service. The report forms cover occupation group, kind of activity and procedure under which the NSI occurred and name of the index patient. Contact of body fluids with skin or mucous membranes were not considered in our study. Usually, markers for hepatitis B virus (HBV), hepatitis C virus (HCV) and human immunodeficiency syndrome (HIV) were determined for the index patient and injured HCP. We investigated all medical disciplines and all occupational groups, including students, at our university hospital.

In 2008 safety devices were introduced throughout the hospital including all departments and all operating rooms, e.g. peripheral venous catheter, hypodermic needle and stapling system for wound sealing providing active or passive protection. In our study, the safety mechanism was triggered actively for hypodermic needles and butterfly systems, a passive mechanism was available for peripheral venous catheters, lancets and port needles. Additionally, training was performed in all departments of the Heidelberg University Hospital by the Occupational Health Service and was obligatory for all healthcare personnel in 2008 when the new safety devices were introduced. The chi-square test was applied for all statistical calculations in order to compare the number of needlestick injuries before and after the introduction of safety devices. A p-value below 0.05 was regarded as statistically significant.

## Results

In 2007, altogether 448 NSIs were self-reported. In 2009, one year after the introduction of safety devices in all departments, a 21.9% decline of NSIs was observed in HCP resulting in 350 NSIs. The annual rate of NSIs in 2007 was 69.0 per 1 000 full-time HCP and dropped to an annual rate of 52.4 per 1 000 full-time HCP in 2009. Infection with HBV, HCV or HIV in needlestick index patients was high in both years, with a prevalence of 6.7% in 2007 and 9.0% in 2009 for all three bloodborne viruses together. In both years, about 5% of the needlestick-injured employees had an insufficient anti-HBs titer (data not shown).

Of all occupational groups, nurses (36.2%) had the highest risk to experience NSI, followed by medical doctors with 33.7% in 2007 (Table 1). The same phenomenon was still observed in 2009, however a significant decrease in the number of NSIs was detected. No significant difference was observed for medical students after the introduction of safety devices, 72 vs. 65 in 2007 and 2009 respectively (p-value 0.300). The highest incidence of needlestick injuries in 2007 was observed among medical staff in the surgery department and internal medicine with 152 (33.9%) and 79 (17.6%) NSIs, respectively, followed by a decline in 2009 to 119 (34.0%) and 65 (18.6%) NSIs (Table 2).

**Table 1:** Number and percentage of reported needlestick injuries in 2007 and 2009 within different professional groups

	**NSIs 2007 N (%)**	**NSIs 2009 N (%)**	**p-value**
nurses	162 (36.2%)	127 (36.3%)	0.008
medical doctors	151 (33.7%)	114 (32.6%)	0.003
students	72 (16.1%)	65 (18.6%)	0.300
laboratory	16 (3.6%)	9 (2.6 %)	0.145
others	47 (10.5%)	32 (9.1%)	0.025
total	448 (100%)	350 (100%)	

**Table 2:** Number and percentage of reported needlestick injuries in 2007 and 2009 within different departments of the Heidelberg University Hospital

	**NSIs 2007 N (%)**	**NSIs 2009 N (%)**	**p-value**
surgery	152 (33.9%)	119 (34.0%)	0.002
internal medicine	79 (17.6%)	65 (18.6%)	0.048
gynaecology	45 (10.0%)	37 (10.6%)	0.224
dentistry	41 (9.2%)	22 (6.3%)	0.007
anaesthesisiology	32 (7.1%)	13 (3.7%)	0.005
pediatrics	16 (3.6%)	26 (7.4%)	0.154
psychiatry	12 (2.7%)	3 (0.9%)	0.015
neurology	11 (2.5%)	8 (2.3%)	0.275
radiology	7 (1.6%)	9 (2.6%)	0.700
others	11 (2.5%)	14 (4.0%)	0.759
total	448 (100%)	350 (100%)	

A wide variation of the number of NSIs was found for different instruments applied or performed procedures (Table 3). An increase in the number of NSIs was determined for the devices port needle and lancet in 2009. However, after the introduction of safety devices, a clear decrease in the number of NSIs was observed for stapling systems used for wound sealing, blood withdrawal, hypodermic needles and peripheral venous catheter in 2009. Taken together, the number of NSIs decreased overall by 21.9% after the introduction of safety devices. The highest reduction for NSIs was found when safety devices were applied for blood withdrawal, peripheral venous catheter and hypodermic needles.

**Table 3:** Applied device or performed procedure during needlestick injury and number of NSIs in 2007 and 2009

	**NSIs 2007 (N)**	**NSIs 2009 (N)**	**Decrease/increase 2009 (p-value)**
surgical instrument	102	82	- 19.6% (0.826)
port needle	78	88	+ 12.8% (0.008)
stapling system (wound sealing)	52	35	- 32.7% (0.470)
blood withdrawal	50	27	- 46.0% (0.010)
peripheral venous catheter	49	21	- 57.1% (0.014)
hypodermic needle	47	20	- 57.4% (0.016)
lancet	19	25	+ 31.6% (0.075)
others	51	52	+ 2.0% (0.146)
total	448 (100%)	350 (100%)	- 21.9%

## Discussion

The purpose of this study was to investigate the frequency and cause of needlestick injuries at a university hospital in southwestern Germany before and after hospital-wide introduction of safety devices. The risk of infection by NSIs can be calculated from the number of accidents, the prevalence of active infection in the patient population, the probability of infection after percutaneous exposure and the proportion of HCP susceptible to infection [8]. This risk may be reduced by increasing the vaccination rate against hepatitis B and decreasing the number of NSIs.

The infection rate with bloodborne diseases in needlestick index patients in our study group was 1.7% for HBV, 5.3% for HCV and 2.0% for HIV in 2009. Germany has a low prevalence of bloodborne infections, prevalence for anti-HCV is 0.4 – 0.7%, and 0.05% for HIV in the general population. Thus infections with bloodborne disease of index patients in our study group are about 10 to 40 times higher than in the general German population. Similar results had been reported by Wicker et al [8]. The high rate of patients infected with HBV, HCV or HIV among index patients could be related to a higher reporting of NSIs when patients are known to be infected.

In our study group, 448 NSIs were self-reported in 2007 to the Occupational Health Service, corresponding to an annual rate of 69.0 per 1 000 full-time healthcare personnel. In 2009, when safety devices were used across all disciplines in our hospital, the annual rate of NSIs decreased to 52.4 per 1 000 HCP. The annual rate of NSIs in Germany in hospitals is 29.9 per 1 000 full-time HCP, versus 7.4 for all other HCP outside hospitals [9]. Other studies mostly used an anonymous questionnaire or online survey on a voluntary basis. These studies found higher rates of NSIs, ranging from 22% to 59% [3,10-12]. Nonreporting rates between 45% and 75% have been published recently [11-15]. Students had nearly twice the number of NSIs compared with dentists with ten years working experience according to Wicker and Rabenau [16]. In our University Hospital, students have less experience in error-prone procedures and training is less intense when compared to nurses and medical doctors. Involvement in bloodwithdrawals and surgical procedures increased drastically over the last years, this might explain the unchanged number of NSIs for students.

A wide variation in the number of reported NSIs was evident across disciplines, the highest incidence was observed among medical staff in the surgery department and internal medicine. Wicker et al. have analysed some disciplines at a university hospital and reported also the highest incidence of NSIs in the department of surgery [17]. While accidental NSIs were most frequent in surgery, the nominal risk of blood-borne virus infection was greatest in the field of internal medicine due to increased prevalence of blood-borne pathogens in patients under treatment in this study [8]. Of all occupational groups, nurses had the highest risk to experience NSI, followed by medical doctors at our hospital. In contrast, the share of physicians was highest (55.8%), followed by nurses (22.2%) at the university hospital of Frankfurt, Germany [17]. Luthi et al. reported physicians having the highest risk within all occupational groups [18].

The best way to protect against NSI is use of safety devices. These devices are a suitable and important tool in the reduction of NSIs, and the implementation of safety devices should result in an improvement in medical staff’s health and safety [19,20]. In our study, an overall reduction of 21.9% for NSIs was achieved when safer devices, e.g. peripheral venous catheter, hypodermic needle and stapling system for wound sealing providing active or passive protection, were applied. The number of NSIs was reduced by even 50% for blood withdrawal, use of peripheral venous catheters and application of hypodermic needles. Safety devices need to fulfil criteria as a recognised technical standard, e.g., should be easy to activate, intuitive to use, do not hinder use, and have clear awareness of activation. In our study, the safety mechanism was triggered actively for hypodermic needles and butterfly systems, a passive mechanism was available for peripheral venous catheters, lancets and port needles. It might be speculated that safety devices were sometimes not adequately activated or used not properly, resulting in no overall further decrease in the number of NSIs. According to Butsashvili et al., the most frequent risk for receiving a cut was related to a false move during a procedure, reassembling devices and handing devices [21]. They reported the highest proportion of NSIs among physicians (22%) and nurses (39%) as related to recapping of needles. In a study of Salzer et al., 28% of respondents did not follow established safety regulation such as accurate disposal of contaminated needles [12]. Safety devices are available for about one third of NSI sustained in all medical procedures and most HCP reported being satisfied with anti-needlestick devices [14]. A frequent argument against safety devices is the higher price compared to conventional sharps. On average, about 50% of all NSIs could have been avoided by the use of safety devices, whereas only 13% could have been prevented by organizational measures [17]. This emphasizes that organisational measures are not sufficient and implementation of technical measures is required. The advantage of protective devices for catheter systems to reduce incidence of NSI had been reported [22] and was confirmed in our study as demonstrated by reduction of NSIs by more than half. An implementation of the safety-Lok resheatable winged steel needle in a hospital in New York City demonstrated that NSIs declined by half [23]. The highest proportion of occupational transmission is due to percutaneous injury via hollow-bore needles with vascular access [24].

This study refers to self-reported NSIs, the rate of self-reported NSIs among HCP might have varied in 2007 and 2009. Thus a variation in the rate of reporting cannot be exluded. However, during the last decade, a continuously increasing reporting rate was observed at our university hospital and argues against a significant variation in the reporting rate. The study did not cover degree of extended work shifts, time pressure or under-staffing influencing the number of needlestick infections. For pediatric patients, only a limited amount of safety devices with minimum loss of blood is available at the moment.

In conclusion, there is a high rate of NSI in the daily routine of a hospital, the rate of such injuries depends primarily on the medical discipline. Implementation of safety devices led to an improvement in medical staff’s safety.

## Competing interests

The authors declare that they have no conflict of interest.

## Authors’ contributions

CH, LB and PS collected and analysed the data. PS drafted the manuscript. All authors read and approved the final manuscript.
